# MiR-188-5p regulates the proliferation and differentiation of goat skeletal muscle satellite cells by targeting calcium/calmodulin dependent protein kinase II beta

**DOI:** 10.5713/ab.23.0085

**Published:** 2023-06-26

**Authors:** Jing Jing, Sihuan Zhang, Jinbo Wei, Yuhang Yang, Qi Zheng, Cuiyun Zhu, Shuang Li, Hongguo Cao, Fugui Fang, Yong Liu, Ying-hui Ling

**Affiliations:** 1 College of Animal Science and Technology, Anhui Agricultural University, Hefei 230036, China; 2 Anhui Province Key Laboratory of Local Livestock and Poultry Genetic Resource Conservation and Bio-Breeding, Anhui Agricultural University, Hefei 230036, China; 3 Key Laboratory of Embryo Development and Reproductive Regulation of Anhui Province, Fuyang Normal University, Fuyang, Anhui Province 236041, China

**Keywords:** *CAMK2B*, Goat, miR-188-5p, Myogenic Differentiation, Myogenic Proliferation, Skeletal Muscle Satellite Cells

## Abstract

**Objective:**

The aim of this study was to reveal the role and regulatory mechanism of miR-188-5p in the proliferation and differentiation of goat muscle satellite cells.

**Methods:**

Goat skeletal muscle satellite cells isolated in the pre-laboratory were used as the test material. First, the expression of miR-188-5p in goat muscle tissues at different developmental stages was detected by quantitative reverse transcription polymerase chain reaction (qRT-PCR). In addition, miR-188-5p was transfected into goat skeletal muscle satellite cells by constructing mimics and inhibitors of miR-188-5p, respectively. The changes of differentiation marker gene expression were detected by qPCR method.

**Results:**

It was highly expressed in adult goat latissimus dorsi and leg muscles, goat fetal skeletal muscle, and at the differentiation stage of muscle satellite cells. Overexpression and interference of miR-188-5p showed that miR-188-5p inhibited the proliferation and promoted the differentiation of goat muscle satellite cells. Target gene prediction and dual luciferase assays showed that miR-188-5p could target the 3′untranslated region of the calcium/calmodulin dependent protein kinase II beta (*CAMK2B*) gene and inhibit luciferase activity. Further functional studies revealed that *CAMK2B* promoted the proliferation and inhibited the differentiation of goat muscle satellite cells, whereas *si-CAMK2B* restored the function of miR-188-5p inhibitor.

**Conclusion:**

These results suggest that miR-188-5p inhibits the proliferation and promotes the differentiation of goat muscle satellite cells by targeting *CAMK2B*. This study will provide a theoretical reference for future studies on the molecular mechanisms of skeletal muscle development in goats.

## INTRODUCTION

Skeletal muscle is the most abundant tissue in mammals and has a remarkable regenerative capacity [1,2]. Its formation is a complex process, including the recruitment of myogenic precursors into myocytes, myoblast proliferation, cell cycle arrest, and the fusion of muscle cells to form multinucleated muscle fibers. Some studies have shown that skeletal muscle production involves the transcriptional and epigenetic myogenic regulators [3]. At the transcriptional level, myogenic regulatory factors (MRFs) including myogenic differentiation (*MYOD1*), myogenin (*MYOG*), myogenic regulator (*MRF4*), and myogenic factor (*Myf5*) are required for myogenic progression [4]. Activation of skeletal muscle satellite cells (SMSCs) depends on the upregulated expression of *Myf5* and *MYOD1*, including the combined and coordinated effects of multiple intrinsic and extrinsic factors as well as other cell types cells [5,6]. These factors collaborate with *MRF4* and *MYOG* to regulate the development of SMSCs. As a downstream gene of MYOD1, MYOG is a marker of SMSCs differentiation and is also closely related to the activation of certain other genes during this process as its knockout suppresses the differentiation of muscle cells, and blocks the fusion of myotubes [7]. These myogenic regulators collectively facilitate muscle growth and development [8]. Hence, a further understanding of these factors is required to increase the existing knowledge of skeletal muscle development, which will in turn help to facilitate mutton goat breeding and improve meat quality.

MicroRNAs (miRNAs) are endogenously expressed small noncoding RNAs of 19 to 25 nucleotides in size that regulate the post-transcriptional silencing of target genes [9]. Many studies have now demonstrated that these factors participate in skeletal muscle differentiation. The muscle-specific miRNAs, miR-206, miR-1, and miR-133, are abundantly expressed following the inhibition of specific transcription repressors during skeletal muscle typing, proliferation and differentiation [10,11]. In addition, many non-muscle-specific miRNAs also regulate muscle proliferation and differentiation through post-transcriptional mechanisms, which positively or negatively affect the existence and function of myogenic factors [12]. Further study of functional miRNAs in goat muscle development remains necessary to improve our understanding of how the miRNA network regulates goat myogenesis.

Our previous study explored the expression profile of miRNAs in goat skeletal muscle at different stages [13], in which miR-188-5p was found to be differentially expressed between a goat fetus at 120 days (F120) and 135 days (F135). Notably, muscle cells are at their peak of proliferation and differentiation prior to 120 days of gestation. Previous studies have shown that although miR-188 is not muscle-specific miRNA, its expression in skeletal muscle is up-regulated. At the same time, through the *in vitro* analysis of C2C12, it was found that miR-188 may be involved in the process of myogenic differentiation of cells [14]. It was also found that miR-188-3p could regulate the proliferation and differentiation of vascular smooth muscle cells [15]. Therefore, we speculate that miR-188-5p may be involved in the process of goat muscle development.

Goat meat is an advantageous food for the national market as a product with low fat, low cholesterol content and high-quality protein. China is abundant in goat breed resources and has a considerable number of goats. There are also large number of local high-quality breeds, which have tender meat and their own characteristics, and some of them have excellent meat quality but low meat production performance, including the local breed Anhui White Goat that we studied. Anhui white goat is an excellent local goat breed in Anhui Province, which has the advantages of high fecundity, rough feeding tolerance and delicious meat quality, but its meat production performance is not high. In order to improve its meat production performance, we studied its muscle development process. However, few studies have reported the characterization of miR-188-5p target genes and regulatory networks in muscle cells. Therefore, the aim of our study is to assess the expression profiles of miR-188-5p in various Anhui white goat tissues and investigate its effects on proliferation and differentiation of primary myoblasts.

## MATERIALS AND METHODS

### Animal and cell culture

Twelve fetuses (60, 90, 120, and 135 days), 6 kids (1 and 90 days old), and 3 adult males of the Anhui white goat variety were used for expression profiling and were purchased from the Hefei Boda Company (Hefei, China). The animal management and sample collection protocols used have been described in detail in our previous study [16]. Subsequently, the samples were immediately snap frozen in liquid nitrogen and then stored at −80°C for further experiments. The heart, liver, spleen, lung, kidney, longissimus dorsi and leg muscles of the adult male goats were collected and preserved with this same method. All surgical procedures involving animals were conducted in accordance with the NIH guidelines for the care and use of laboratory animals (http://grants1.nih.gov/grants/olaw/references/phspol.htm). The procedures involving goats had been given prior approval by the ethics committee of Anhui Agricultural University under permit No. AHAU20101025.

Primary goat SMSCs were isolated from the longissimus dorsi tissues of a lamb and were purified and were purified and cultured according to a previously described protocol [7]. SMSCs were cultured in DMEM/F12 basic medium (HyClone, Logan, UT, USA) supplemented with 10% fetal bovine serum (FBS; Gibco, Grand Island, NY, USA) and 1% penicillin-streptomycin solution (100 U/mL penicillin and 100 μg/L streptomycin; Beyotime, Shanghai, China) at a constant at 37°C in a 5% CO_2_ humidified atmosphere. For myoblast differentiation the standard growth media was replaced with differentiation medium (DMEM/F12 basic medium containing 2% FBS).

### RNA Isolation and quantitative reverse transcription polymerase chain reaction

For quantitative reverse transcription polymerase chain reaction (qRT-PCR), total RNA was extracted from goat tissue samples or cultured cells using Trizol reagent (Takara, Dalian, China). The purity and concentration of these RNA samples were evaluated using a NanoDrop2000 spectrophotometer (Thermo, San Jose, CA, USA). Standard denaturing agarose gel electrophoresis was used to detect contamination and degradation. Reverse transcription was conducted using about 1 μg of total RNA using the ReverTra Ace qPCR RT Master Mix, which includes a gDNA remover (Toyobo, Shanghai, China). The generated cDNAs were stored at −20°C for subsequent usage. Gene expression levels were then detected by qPCR using the SYBR Green Kit (Tolo Biotech, Shanghai, China) on a Stepone qPCR detection system (ABI). The primer sequences are listed in Table 1 and those for *MYOD1*, *MYOG*, *MyHC*, calcium/calmodulin dependent protein kinase II beta (*CAMK2B*), and glyceraldehyde-3-phosphate dehydrogenase (*GAPDH*) were designed using primer-BLAST from NCBI (https://www.ncbi.nlm.nih.gov/tools/primer-blast/index) and synthesized by General Biologicals (Anhui, China). The primers for miR-188-5p and U6 small nuclear RNA (*U6*) were designed and synthesized by Guangzhou RiboBio Co, Ltd. (Guangzhou, China).

### Target prediction and dual-luciferase reporter assay

To explore the molecular mechanisms of miR-188-5p functions during goat myoblast development, the potential target genes for miR-188-5p were predicted using the online software platforms TargetScan (http://www.targetscan.org/vert_80/), RNAhybrid (https://bibiserv.cebitec.uni-bielefeld.de/rnahybrid/) and miRanda v3.3a. Wild type and mutant partial length sequences (containing miR-188-5p seed sequence target sites) of the *CAMK2B*, 3′untranslated region (3′UTR) were cloned into the psi-Check2 vector with T4 DNA ligase (Takara Bio Inc. Kusatsu, Shiga, Japan), and confirmed by sequencing (RiboBio, China). 293T cells were then seeded onto 6-well plates and maintained in 1,640 medium (DMEM) (Gibco, USA) containing 10% FBS (Gibco, USA) at 37°C and 5% CO_2_ in a humidified atmosphere. These plasmids and the miR-188-5p mimic were co-transfected into 293T cells separately. Twenty-four hours after transfection, the cells were lysed and analyzed using a Dual-Luciferase Reporter Assay System (Promega, Madison, WI, USA).

### Vector construction, mimics, inhibitor, and siRNA synthesis

The miR-188-5p mimic, inhibitor (CCCUCCACCAUGCA AGGGAUG) and an *CAMK2B* siRNA (Sense: CCAGCUC UACGAGGAUAUUTT; Antisense: AAUAUCCUCGUAG AGCUGGTT) were synthesis in Gene Pharma (Shanghai, China).

### Cell transfection

Prmary goat adult myoblasts were cultured in 12-well plates for 18 to 24 hours until cell density reached 70% to 80% confluence prior to transfection. miR-188-5p overexpression and knockdown were achieved by transfecting adult myoblasts with miRNA mimics or inhibitors, respectively. TrLipofectamine 2000 reagent (Thermo Fisher Scientific, Waltham, MA, USA) was used to transfect goat SMSCs with these constructs in accordance with the manufacturer’s specifications. All experiments were performed in three independent experiments with at least three replicates. Finally, cells were harvested for RNA and protein extraction 48 hours after transfection.

### EdU proliferation assay

Satellite cell proliferation was measured via an EdU cell proliferation assay kit in accordance with the manufacturer’s protocol (RiboBio, China). Briefly, goat skeletal muscle SMSCs were seeded onto 96-well plates (3,000 cell/well) and cultured in growth medium to a normal growth stage after transfection. The medium was then removed, and the cells were washed with phosphate buffered saline (PBS) and incubated for 8 hours with medium containing 100 μL EdU. Immunostaining was then performed, and the fluorescence values were recorded using a GE IN CELL, a 6500HS high-content analyzer and the data were analyzed with ImageJ software. The ratio of EdU-positive cells was calculated as follow: (EdU-positive cells/DAPI-stained cells)×100%.

### Protein extraction and western blotting analysis

Total proteins were extracted using 99% RIPA lysis buffer (TaKaRa, China) containing 1% phenylmethylsulfonyl fluoride (Beyotime, China) after washing the cells with PBS three times. The protein concentration was then determined using the BCA Protein Assay Kit (Beyotime, China; and 30 μg aliquots were separated by 12% sodium dodecyl sulfate polyacrylamide gel electrophoresis followed by transfer onto a polyvinylidene fluoride membrane (Servicebio, Wuhan, China). The membrane was blocked with 5% defatted milk in Tris-buffered saline with Tween 20 (TBST) buffer for 1 h at room temperature, and then incubated with antibodies (1:1,000) against CAMK2B, and GAPDH (1:2,000; CUSABIO Life Sciences, College Park, MD, USA) at 4°C overnight. The following day, each membrane was washed three times with TBST for 10 min each time and then incubated with HRP-conjugated secondary antibodies (anti-mouse IgG) (Abcam, Cambridge, UK) diluted 1:2,000. Chromogenic reactions were performed using an ECL western blot substrate (Solarbio, Shanghai, China) and the signals were quantified with the ChemiDoc XRS+ system (Bio-Rad, Hercules, CA, USA).

### Bioinformatics

The TargetScan (http://www.targetscan.org/vert_80/), RNAhybrid (https://bibiserv.cebitec.uni-bielefeld.de/rnahybrid/) and miRanda v3.3a platforms were used to predict binding sites.

### Statistical analysis

All data were expressed as the means±standard error and were compared by one-way analysis of variance and T test with SPSS V25.0. Differences were regarded as significance at p<0.05.

## RESULTS

### Tissue and muscle satellite cell expression profiles of miR-188-5p

**Figure 1:**
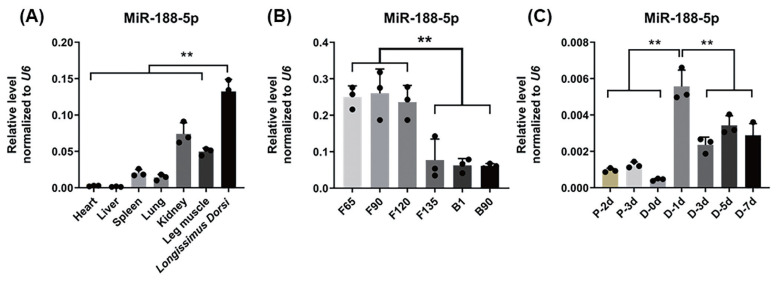
Tissue and muscle satellite cell expression profiles of miR-188-5p. (A) Tissue expression profile of miR-188-5p in different tissues of Goat of fetal lambs at 120 days of gestation. (B) Expression profile of miR-188-5p at different developmental stages in longissimus dorsi muscle of goat. (C) Expression of miR-188-5p in cell proliferation and differentiation. Data were the mean and standard errors (n = 3). F, fetal stage; B, born. P, proliferation phase; D, differentiation period. ** p<0.01. The closed points in the graph are the sizes of the three sample values in the experimental group.

To explore the functions of miR-188-5p in muscle tissue and SMSCs in the goat, the expression pattern of this miRNA was examined in different tissues of fetal lambs at 120 days of gestation in different types of muscle tissues and SMSCs at different stages by qRT-PCR. In the different tissue expression profiles of adult goats, miR-188-5p showed high expression in the longissimus dorsi muscle and leg muscle (p<0.01; Figure 1A). The data showed that miR-188-5p is enriched in skeletal muscle over first 65, 90, and 120 days of gestation in the goat fetus and that after 135 days, this expression level showed a significant decline (p<0.01; Figure 1B). Furthermore, we observed that miR-188-5p had a high expression level at day 1 of muscle satellite cell differentiation but gradually decreased thereafter (p<0.01; Figure 1C). These results suggested that miR-188-5p might play a regulatory role at both the fetal and early differentiation stages of skeletal muscle development in the goat.

### miR-188-5p inhibits the proliferation of muscle SMSCs and promotes their differentiation

**Figure 2:**
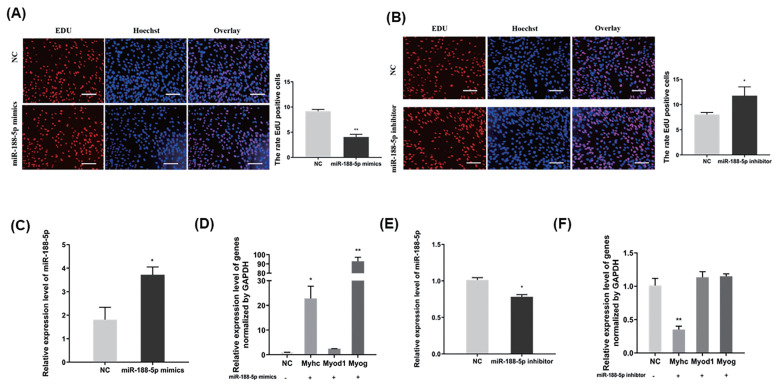
Inhibition of the proliferation and promotion differentiation of SMSC by miR-188-5p. (A) Detection of cell proliferation efficiency by EdU after miR-188-5p mimic. Scale bar, 100 μm (B) Detection of cell proliferation efficiency by EdU after miR-188-5p inhibitor. Scale bar, 100 μm (C) miR-188-5p mRNA expression detected by qRT-PCR in goat myoblasts transfected with miR-188-5p mimics or NC. (D) qRT-PCR analyses of the expression of differentiation-related genes (*MYOG*, *MYOD1*, and *MyHC*) in goat myoblasts transfected with miR-188-5p mimics. (E) miR-188-5p mRNA expression detected by qRT-PCR in goat myoblasts transfected with miR-188-5p inhibitors or NC. (F) qRT-PCR analyses of the expression of differentiation-related genes (*MYOG*, *MYOD1*, and *MyHC*) in goat myoblasts transfected with miR-188-5p inhibitors. Data were the mean and standard errors (n = 3). SMSC, skeletal muscle satellite cells; qRT-PCR, quantitative reverse transcription polymerase chain reaction; *MYOG*, myogenin; *MYOD1*, myogenic differentiation 1; *MyHC*, myosin heavy chain 1. * p <0.05 and ** p<0.01 as compared with negative control.

To confirm the underlying effects of miR-188-5p on SMSCs regulation, we tested the role of miR-188-5p in the proliferation and differentiation of these cells. EdU incorporation assay demonstrated in this regard that miR-188-5p overexpression significantly inhibited myoblast proliferation (Figure 2A). By contrast, the anti-miR-188-5p group showed a significant promotion of myoblast proliferation (Figure 2B). Transfection of SMSCs with miR-188-5p mimics led to the significantly increased expression of the differentiation-related *MYOG* and *MyHC* genes (Figure 2C, D). The expression of miR-188-5p was successfully suppressed by transfection of a specific inhibitor (Figure 2E). qPCR also showed that the gene expression of *MyHC* in SMSCs was also inhibited following this transfection, whereas the gene expression of *MYOG* and *MYOD1* was unchanged (Figure 2F). Therefore, we showed that overexpression of miR-188-5p inhibited the proliferation of SMSCs, while inhibition of miR-188-5p promoted the proliferation of SMSCs. Meanwhile, miR-188-5p promoted the differentiation of SMSCs.

### *CAMK2B* is a novel target gene of miR-188-5p in SMSCs

**Figure 3:**
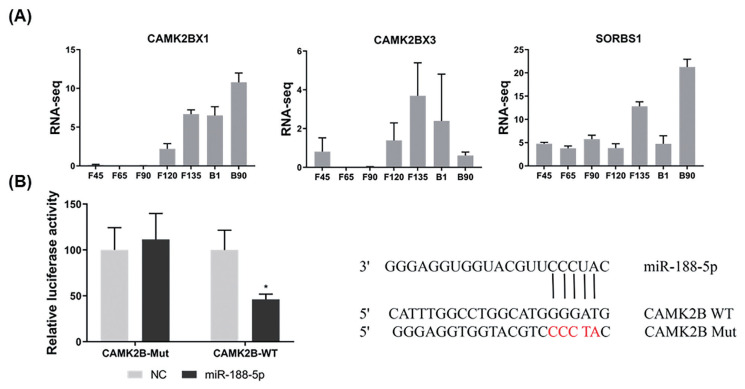
Identification of *CAM2KB* as a novel miR-188-5p target. (A) Expression levels of three target genes predicted at different growth stage (F45 F65 F90 F120 F135 B1 and B90) in goats. (B) Confirmation of *CAM2KB* as a miR-188-5p target by double luciferase assay. Cells were co-transfected with the dual-luciferase reporter containing the wild type (WT) or mutant *CAMK2B* 3′UTR, either with the negative control or miR-188-5p mimics. The activity of renilla luciferase was normalized to firefly luciferase. Data were the mean and standard errors (n = 3). *CAM2KB*, calcium/calmodulin dependent protein kinase II beta. * p<0.05 and ** p<0.01 as compared with negative control.

To explore the molecular mechanisms of miR-188-5p function on goat myoblast development, its potential target genes were predicted using the online software platforms TargetScan, RNAhybrid, and miRanda. Only three target genes were predicted in this software. The mutant *CAMK2BX1*, mutant *CAMK2BX3*, and *SORBS1* genes were thereby identified as candidates for further study. According to previous RNA-seq data [16], only the expression trends for the mutant *CAMK2BX1* (referred to as *CAMK2B*) were opposite to those of miR-188-5p during the 7 developmental stages in goats (Figure 3A). The relative luciferase activity of the wild type 3′UTR group (WT-*CAMK2B*-3′UTR) was significantly inhibited as they responded to miR-188-5p mimics (p<0.01; Figure 3B), while no changes were observed in cells co-transfected with the mutated reporter (Mutant-*CAMK2B*-3′UTR) (Figure 3B). These results suggest the direct target relationship between *CAMK2B* and miR-188-5p. We thus next explored whether *CAMK2B* has a regulatory role in goat SMSCs.

### *CAMK2B* promotes the proliferation of goat muscle SMSCs and inhibits their differentiation

**Figure 4:**
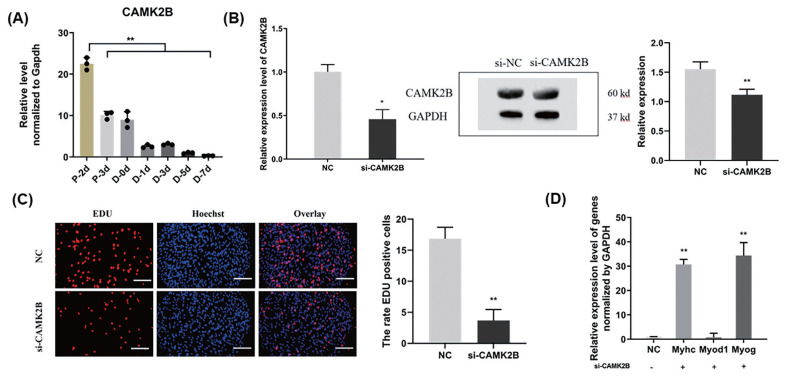
Promotion of the cell proliferation and inhibition of differentiation of muscle SMSCs by *CAMK2B*. (A) The expression of CAMK2B in proliferating and differentiated goat muscle cells. (B) Knock-down verification of mRNA and protein levels of *CAMK2B* gene. (C) EdU staining of goat muscle cells after *si-CAMK2B* treatment. Scale bar, 200 μm (D) qRT-PCR analysis of the differentiation-related genes (*MYOG*, *MYOD1*, and *MyHC*). Data were the mean and standard errors (n = 3). SMSC, skeletal muscle satellite cells; *CAM2KB*, calcium/calmodulin dependent protein kinase II beta; qRT-PCR, quantitative reverse transcription polymerase chain reaction. * p<0.05 and ** p<0.01 as compared with negative control.

We further examined the role of *CAMK2B* during goat muscle SMSCs proliferation and differentiation using RNA interference. We found in the first instance that the *CAMK2B* gene is highly expressed in SMSCs in the proliferative phase and decreases suddenly during differentiation (p<0.01; Figure 4A). Compared with a si-NC control, the expression of CAMK2B was blocked at both the mRNA and protein levels (Figure 4B), and the proliferation rate of SMSCs was significantly reduced after transfection with a *si-CAMK2B* construct (Figure 4C). We thus examined the impact of a *CAMK2B* knockdown via RNAi on myoblast differentiation. Additional qPCR analysis revealed that the gene expression of *MyHC* and *MYOG* was significantly upregulated in differentiated SMSCs, while the expression of *MYOD1* was unhanged (Figure 4D). These results indicated that *CAMK2B* affects the proliferation and differentiation of SMSCs in an opposite manner to the regulatory function of miR-188-5p.

### miR-188-5p regulates the proliferation and differentiation of muscle SMSCs through *CAMK2B*

**Figure 5:**
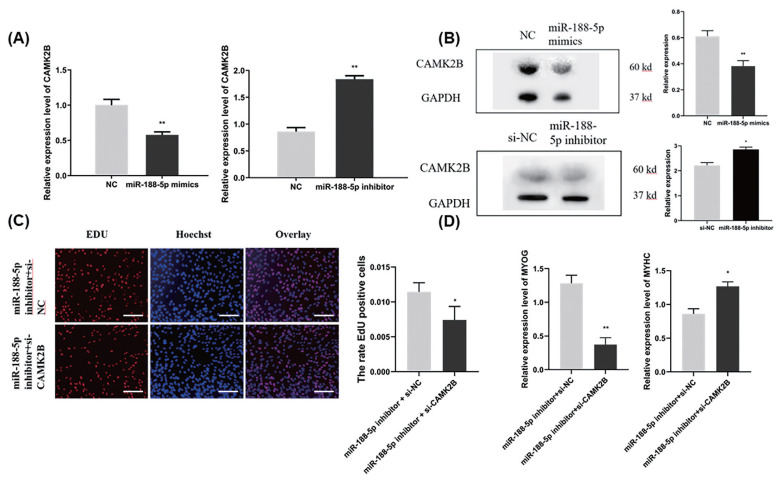
Regulation of proliferation and differentiation of SMSCs by miR-188-5p through *CAMK2B*. (A) The mRNA expression level of *CAMK2B* gene after overexpression or inhibition of miR-188-5p (B) The protein expression level of *CAMK2B* after overexpression and inhibition of miR-188-5p. (C) EdU staining of SMSCs after *si-CAMK2B* and miR-188-5p inhibitor transfection. Scale bar, 100 μm (D) The expression level of *MyHC* and *MYOG* after co-transfection with *si-CAMK2B* and miR-188-5p inhibitor. Data were the mean and standard errors (n = 3). SMSC, skeletal muscle satellite cells; *CAM2KB*, calcium/calmodulin dependent protein kinase II beta; qRT-PCR, quantitative reverse transcription polymerase chain reaction. * p<0.05 and ** p<0.01 as compared with negative control.

**Figure 6:**
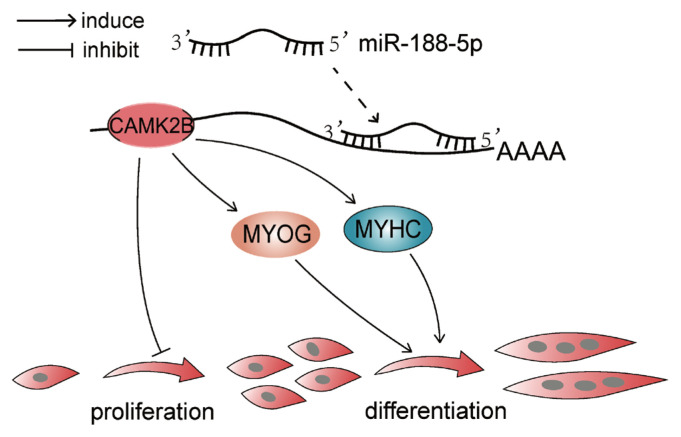
Schematic model of miR-188-5p regulation in myogenesis.

To determine whether the regulation of *CAMK2B* contributed to the inhibition of myogenesis via miR-188-5p, we investigated the regulatory relationship between them in SMSCs. The gene expression of *CAMK2B* was significantly down- or upregulated by the overexpression or inhibition of miR-188-5p, respectively (Figure 5A). We then tested the expression level of the *CAMK2B* protein product after the overexpression or inhibition of miR-188-5p (Figure 5B) and observed a clear negative regulatory relationship between them. Subsequently, *si-CAMK2B* was co-transfected with the miR-188-5p inhibitor to elucidate their interaction. Subsequent EdU results indicated that there was no effect of this co-transfection on SMSCs proliferation (Figure 5C). The expression of the differentiation-related gene *MyHC* was higher however, while that of *MYOG* was decreased (Figure 5D), which was consistent with our earlier observations. These results further indicated that miR-188-5p regulates the proliferation and differentiation of SMSCs by targeting *CAMK2B* (Figure 6).

## DISCUSSION

We have revealed in our present study that miR-188-5p can inhibit the proliferation of SMSCs and promote the differentiation process in these cells. Although miR-188-5p is a muscle non-specific miRNA, the RT-qPCR results for it expression in seven different goat tissues revealed a high abundance in the longissimus dorsi muscle. It showed a significantly differential expression before and after the F120 and F135 developmental timepoints in the goat fetus, which was consistent with previous RNA-seq data [13]. miR-188-5p has been sound to have a variety of regulatory effects in many disorders, including cell proliferation and differentiation [17,18], apoptosis [19], development [20,21], and the onset of various diseases [22]. This miRNA also promotes apoptosis and inhibits proliferation in breast cancer cells via the MAPK signaling pathway by targeting Rap2c [23]. The overexpression of miR-188-5p also inhibits the proliferation of hepatocellular carcinoma cells [24]. In addition, this miRNA has shown an association with osteogenesis and adipogenesis processes and regulates the balance between the bone and fat differentiation pathways of mouse bone marrow stromal stem cells (mBMSCs) [1]. It directly regulates LCoR in mBMSCs, and when miR-188 is overexpressed and LCoR is inhibited, the osteogenic process of these cells is inhibited and adipogenic differentiation is induced [1]. Riaz et al [25] found previously that the inhibition of miR-188-5p alleviates hepatic fibrosis by significantly reducing the activation and proliferation of hepatic stellate cells through the PTEN/PI3K/AKT pathway. However, there have been relatively few studies to date on the involvement of miR-188-5p in skeletal muscle. Some researchers have demonstrated that miR-188-5p promotes oxaliplatin resistance by targeting RASA1 in colon cancer cells [26]. Consistent with its function in other types of cells, miR-188-5p promoted the differentiation and inhibited the proliferation of these cancer cells.

To verify the function of miR-188-5p in goat muscle development in our present study, we used SMSCs to test the effects of this miRNA on the proliferation and differentiation of these cells. By transfecting mimics and inhibitors into the cells, these results showed that miR-188-5p significantly promoted cell differentiation and inhibited proliferation of SMSCs cells, which is consistent with our previous sequencing data [27]. Hiroyuki Shibasaki [14] found miR-188 may participate in the myogenic differentiation of the cells, but suppression of miR-188 expression did not show an effect on the fusion index. It is not quite consistent with our results. After miR-188-5p inhibit, the decrease of MyHC expression may be due to the short days of differentiation and less fusion. But it also proves the importance of miR-188 in the regulation of muscle differentiation. Our findings thus reveal a new function of miR-188-5p in the proliferation and differentiation of goat SMSCs, which goes beyond the effect of this miRNA on cancer [28] and adipogenesis [29].

To further explore the mechanisms underlying the effects of miR-188-5p on proliferation and differentiation, miR-188-5p target genes were predicted using the TargetScan, RNAhybrid and Miranda databases. We identified miR-188-5p target genes that were found previously to be differentially expressed in seven developmental stages in the goat[16]. However, only the *CAMK2B* gene contains binding sites with miR-188-5p, and showed a mutually exclusive expression pattern with it. We found consistently that by increasing the expression of miR-188-5p in SMSCs, the CAMK2B mRNA and protein levels were reduced and vice versa. We further confirmed the targeting relationship between *CAMK2B* and miR-188-5p through dual-luciferase gene reporter assays. The number of SMSCs was also decreased after a knockdown of *CAMK2B* using RNAi, which further indicated that the opposite expression profiles of *CAMK2B* and miR-188-5p. *CAMK2B* is a subtype of the *CAMK2* family, and its role in the brain has attracted widespread attention [30–32]. For example, its splice subtype affects learning and memory in primates [33]. It is mainly associated with calcium-related signaling processes, and its effects on the brain include developmental delays and language loss [34]. Mouse *CAMK2B* mutants exhibit very severe motor deficits, moreover the calcium/calmodulin-dependent activation of *CAMK2B* is essential for normal locomotion [35].

As a potential downstream target of p38 α MAPK, the activity of *CAMK2B* may be related to muscle atrophy and play a certain role in mediating skeletal muscle signal transduction [36]. Previous studies have also found that *CAMK2B* plays an important role in muscle development [37]. In our present study, the number of SMSCs decreased and the expression of differentiation genes *MyHC* and *MYOG* increased significantly after the inhibition of *CAMK2B*. In addition, when we counteracted the effects of *si-CAMK2B* on goat SMSCs proliferation and differentiation by inhibiting miR-188-5p, we found that in addition to the recovery in the SSC numbers, the expression of the differentiation gene *MyHC* was increased, while that of *MYOG* was unaffected. These data thus indicate that miR-188-5p regulates the proliferation and differentiation of goat SMSCs by targeting *CAMK2B*. Our current findings thus provides new insights into the regulation of muscle development in male goats and other male mammals.

## CONCLUSION

MiR-188-5p inhibits the proliferation and promotes the differentiation of goat SMSCs by targeting the *CAMK2B* gene. This finding provides new basic functional data for future research into the regulatory mechanisms of the miRNAs during muscle development. Furthermore, these results provide new insights into the function of miR-188-5p in goat myocytes and will contribute to our understanding of the growth mechanism of goat skeletal muscle.

## Figures and Tables

**Table 1:** Sequences of the primers used for fluorescence quantitative detection

Gene name	Sequence (5′-3′)	Amplicon length (bp)	Ensembl ID
*miR-188-5p*	RT:GTCGTATCCAGTGCAGGGTCCGAGGTA TTCGCACTGGATACGACCCCTCC Forward: CACGCACATCCCTTGCAT Reverse: CCAGTGCAGGGTCCGAGGTA		
*GAPDH*	Forward: CACAGTCAAGGCAGAGAAC Reverse: TACTCAGCACCAGCATCA	108	ENSCHIG00000022516
*U6*	Forward: CTCAGAATCACCCAATGC Reverse: ATGTTCATCCAGTTGTCAC	85	ENSCHIG00000010928
*MYOD1*	Forward: GGAGGAACACTCGCACTTCC Reverse: CCTTGCAGGCCCACAGTAAA	122	ENSCHIG00000031812
*MYOG*	Forward: CGTGGGCGTGTAAGGTGT Reverse: GGCGCTCTATGTACTGGATGG	195	ENSCHIG00000021331
*MYHC*	Forward: CAAGGGTCTACGCAAACACGA Reverse: AGCTTGCGGAATTTGGAGAGG	186	ENSCHIG00000026733
*CAMK2B*	Forward: TTCTCAGTGGGCAGACAGAC Reverse: GTGCTATTCGTCTGGGGCTT	137	ENSCHIG00000022969

*GAPDH*, glyceraldehyde-3-phosphate dehydrogenase; *U6*, U6 small nuclear RNA; *MYOD1*, myogenic differentiation 1; *MYOG*, myogenin; *MYHC*, myosin heavy chain 1; *CAMK2B*, calcium/calmodulin dependent protein kinase II beta.

## Data Availability

Data in the manuscript was unpublished. The datasets analyzed during the current study are available from the corresponding author on reasonable request. If the article is accepted for publication, the data availability statement will be published as part of the accepted article.
